# The genetic advantage of healthy centenarians: unraveling the central role of NLRP3 in exceptional healthspan

**DOI:** 10.3389/fragi.2024.1452453

**Published:** 2024-09-05

**Authors:** Stef F. Verlinden

**Affiliations:** YOXLO B.V., Leiden, Netherlands

**Keywords:** NLRP3, aging, senescence, healthy centenarians, health extension, accelerated aging, auto-aging

## Abstract

Despite extensive research into extending human healthspan (HS) and compressing morbidity, the mechanisms underlying aging remain elusive. However, a better understanding of the genetic advantages responsible for the exceptional HS of healthy centenarians (HC), who live in good physical and mental health for one hundred or more years, could lead to innovative health-extending strategies. This review explores the role of NLRP3, a critical component of innate immunity that significantly impacts aging. It is activated by pathogen-associated signals and self-derived signals that increase with age, leading to low-grade inflammation implicated in age-related diseases. Furthermore, NLRP3 functions upstream in several molecular aging pathways, regulates cellular senescence, and may underlie the robust health observed in HC. By targeting NLRP3, mice exhibit a phenotype akin to that of HC, the HS of monkeys is extended, and aging symptoms are reversed in humans. Thus, targeting NLRP3 could offer a promising approach to extend HS. Additionally, a paradigm shift is proposed. Given that the HS of the broader population is 30 years shorter than that of HC, it is postulated that they suffer from a form of accelerated aging. The term ‘auto-aging’ is suggested to describe accelerated aging driven by NLRP3.

## 1 Introduction

The significant extension of human lifespan (LS) since the 19th century, attributed to advancements in socioeconomic conditions, healthcare, and nutrition (Oeppen and Vaupel, 2002) has propelled individuals into what is sometimes referred to as a ‘red zone.’ In this zone, the incidence of dementia, other disabilities, and frailty increases sharply (Olshansky, 2018). This situation creates a paradoxical effect where curing or preventing one disease not only extends LS but may also inadvertently allow time for the emergence of another, often more debilitating, condition. As a result, this extension of life often leads to individuals living longer but with an increased duration of disability and poor health (Spiers et al., 2021). Currently, the number of healthy life years at birth, or HS, in the Western world is 66.6 years. The duration of life spent in increasingly poor health or disability is approximately 13.5 years, cumulating in an average LS of 80.1 years (Eurostat, 2019; Scott et al., 2021). This realization that we are extending the duration of disease has catalyzed a shift in focus from simply prolonging life to extending health. Therefore, understanding the determinants of HS is crucial. The foremost question is whether these determinants are environmentally influenced or genetically predetermined.

**GRAPH 1:**
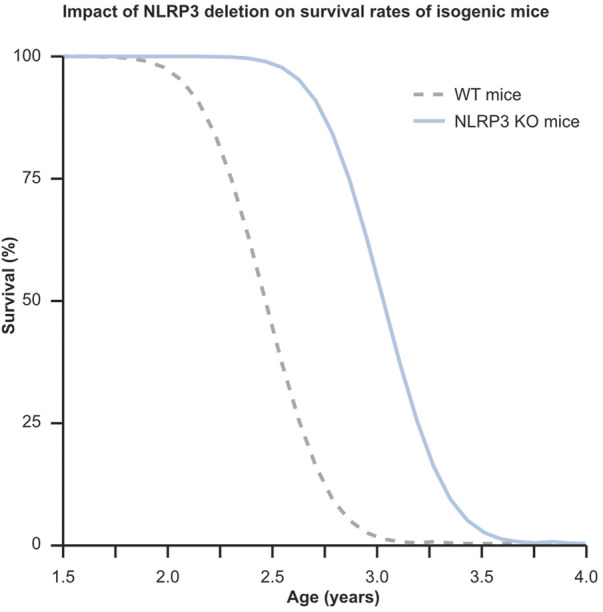
This graph illustrates the profound influence of a single genetic modification on extending lifespan, while also highlighting the stochastic variability in lifespan distribution among genetically identical mice. Despite genetics accounting for only about 25% of individual lifespan variation, the knockout of the NLRP3 gene in a population of mice leads to a notable 32% increase in average lifespan compared to their C57BL wild type counterparts. This underscores the significant potential of targeting aging-related genes responsible for this effect. In the graph, the dotted gray curve represents WT mice, while the solid blue curve depicts NLRP3^−/−^ mice.

Extensive research, including studies on identical twins, suggests that genetics accounts for approximately 25% of the variability in HS and LS (Herskind et al., 1996; Ljungquist et al., 1998). This is further exemplified by the lifespan variations observed in genetically identical C57BL mice, ranging from 13 to 28 months despite uniform genetics and environmental conditions (Marín-Aguilar et al., 2020). These differences underscore the profound impact of environmental variations and stochastic events, such as random cellular mutations and chance encounters with pathogens, on health and longevity and highlight the complex interplay between genetics and the cellular environment. Contrasting these findings, other research shows that single genetic alterations can significantly extend average LS and HS in nematodes, fruitflies, and mice by factors of 2–3, 1.5, and 1.6, respectively. Notably, these genetically modified organisms maintain a similar variability in LS as their wild-type counterparts (Brown-Borg et al., 1996; Wolkow et al., 2000; Clancy et al., 2001). Thus, while genetics account for about 25% of LS variability in genetically identical organisms, a single genetic modification can shift their LS and HS curve to the right (see Graph 1).

The small group of HC exemplifies the possibility for humans to maintain good physical and mental health for at least one hundred years. These remarkable individuals enjoy an extended HS that surpasses the average by over 3 decades. What is more, they typically experience a disease span (morbidity) of approximately half of the 13.5 years of morbidity observed in the general population (Evert et al., 2003; Andersen et al., 2012). The question that arises is: what makes these HC ‘escape’ the diseases that almost everybody else will get? Do they have a beneficial lifestyle and luck that places them at the end of the stochastic distribution curve, or do they possess one or more genetic advantages that protect them from environmental factors that make others sick?

While specific diets seem to increase the chance of becoming a healthy ager, the findings are not entirely consistent. On one hand, studies have found a high adherence to the Mediterranean diet, which is low in animal proteins and carbohydrates, among centenarians (Vasto S et al., 2012). On the other hand, a beneficial effect of a Western-type diet, high in animal protein, fat, and carbohydrates, has been observed among healthy agers over 85 (Gu Q et al., 2020). Additionally, a prospective study of healthy participants over age 80 showed that the chance of becoming a centenarian increased from approximately 1.2% for people adhering to a poor lifestyle to approximately 1.9% for those who strictly adhered to a healthy lifestyle (Li Y et al., 2024). Although this represents a significant effect, the overall contribution of a healthy lifestyle to the likelihood of becoming a centenarian remains relatively minimal.

This minimal effect is reflected in the finding that HC exhibit similarly unhealthy behaviors as individuals in the general population, yet still manage to reach 100 in good health (Rajpathak et al., 2011). Additionally the offspring of these HC tend to remain healthy for significantly longer than those of individuals in the general population. Furthermore, the partners of these offspring, who are likely exposed to similar lifestyle factors, do not experience the same extended HS, suggesting a genetic advantage that protects against aging and accompanying age-related diseases (Perls et al., 2002; Schoenmaker et al., 2006; Sebastiani et al., 2021). This implies that HC do not merely represent the far end of a stochastic distribution curve related to age but have a beneficial genetic trait.

Genetic aberrations that accelerate the onset of common diseases or cause orphan diseases are widely known. Conversely, genetic variants are also known to protect against certain diseases. A notable example is the APOE2 variant of the apolipoprotein E gene, which significantly reduces the risk of acquiring Alzheimer’s disease (Reiman et al., 2020). Emerging evidence suggests that genetic variants leading to altered expression levels or improved versions of specific proteins play a role in the successful aging of HC (Paolisso et al., 1998; Chondrogianni et al., 2000; Alonso-Fernández et al., 2008; Erikson et al., 2016; Tedone et al., 2019). Therefore, it is likely that HC have acquired or inherited one or more of these advantageous traits, meaning that by identifying and harnessing these genetic advantages, innovative treatments could be designed to decelerate or even reverse aging in the broader population. One potential target is the NOD-like receptor family, pyrin domain-containing 3 (NLRP3), which functions as a sensor in the innate immune system and plays a significant role in various age-related pathologies. Chapter 2 provides a detailed discussion of NLRP3’s mechanism of action and involvement in these processes, supported by extensive literature. Humans over the age of 60 exhibit NLRP3 levels that are significantly higher than those in individuals around age 25, whereas HC have been found to maintain NLRP3 expression levels comparable to those of younger individuals, and diseased centenarians exhibit NLRP3 levels that fall between those of HC and younger people (Tedone et al., 2019). Strikingly, the deletion of the Nlrp3 gene in C57BL mice leads to a phenotype akin to that of HC, characterized by delayed onset of bone loss, immunosenescence, frailty, reduced age-related diseases, and a 34% increase in mean HS and LS (Youm et al., 2012; Youm et al., 2013; Marín-Aguilar et al., 2020). These findings support the notion that the exceptional HS of HC is modulated by one or a few critical biological mechanisms, with NLRP3 standing out as a prime candidate for regulation. What is more, when using HC as a benchmark, it becomes evident that the vast majority of individuals who lack this genetic advantage experience accelerated organismal aging, resulting in prolonged morbidity and significantly reduced HS and LS. This review delves into the central role of NLRP3 in the aging process and evaluates its potential as a target to extend human HS.

## 2 The paradoxical role of NLRP3 in health and aging

NLRP3, also known as Cold autoinflammatory syndrome 1 protein (CIAS1), cryopyrin, NACHT, LRR, and PYD domain-containing protein 3 (NALP3), was initially discovered for its role in cryopyrin-associated periodic syndrome (CAPS) (Hoffman et al., 2001). The periodic fever episodes in CAPS patients, in which NLRP3 is overly active, are caused by the strong pyrogen and pro-inflammatory cytokine interleukin (IL)-1β (March et al., 1985). As it turns out, NLRP3 functions as a critical sensor in the innate immune system, triggering a rapid, robust, and complex response against bacterial and viral intruders and in response to tissue damage, thereby preserving health. However, emerging evidence suggests that increased priming and chronic low-grade activation of NLRP3 also play a detrimental role in aging. Therefore, it is crucial to differentiate between its beneficial short-term activation and the potentially detrimental effects of chronic activation. The following sections will thoroughly review and discuss this dichotomy and its implications for health and disease.

### 2.1 Short-lived activation of NLRP3 restores health

NLRP3, an innate immune system receptor, is activated through a specific two-step priming process by distinct pathogen- and damage-associated patterns (PAMPs and DAMPs) or signals of cell stress and damage. NLRP3 is part of a family of pattern recognition receptors (PRRs), which includes membrane-bound Toll-like receptors (TLRs) and cytoplasmic nucleotide-binding and oligomerization domain (NOD)-like receptors (NLRs). NLRP3 and other NLRs can recruit caspases upon activation, forming large multi-protein complexes known as inflammasomes. These complexes produce interleukin (IL)-1β and IL-18, crucial for initiating inflammatory responses and pore-forming N-terminal fragments (Nt) of gasdermin D (GSDMD). This leads to pyroptosis and the subsequent clearance of the activation signal, thereby restoring homeostasis and health (Martinon et al., 2002; Shi et al., 2015). NLRP3 plays a unique role within inflammasome-forming NLRs. Its activation leads to pyroptosis, a lytic and highly inflammatory form of programmed cell death. This process significantly contributes to the innate immune response by facilitating the rapid clearance of pathogens and damaged cells. As inflammasomes induce pyroptosis and inflammation with potentially catastrophic results, they are tightly regulated through a two-step process of priming and activation (D’souza and Heitman, 2001).

#### 2.1.1 Priming of NLRP3

**FIGURE 1:**
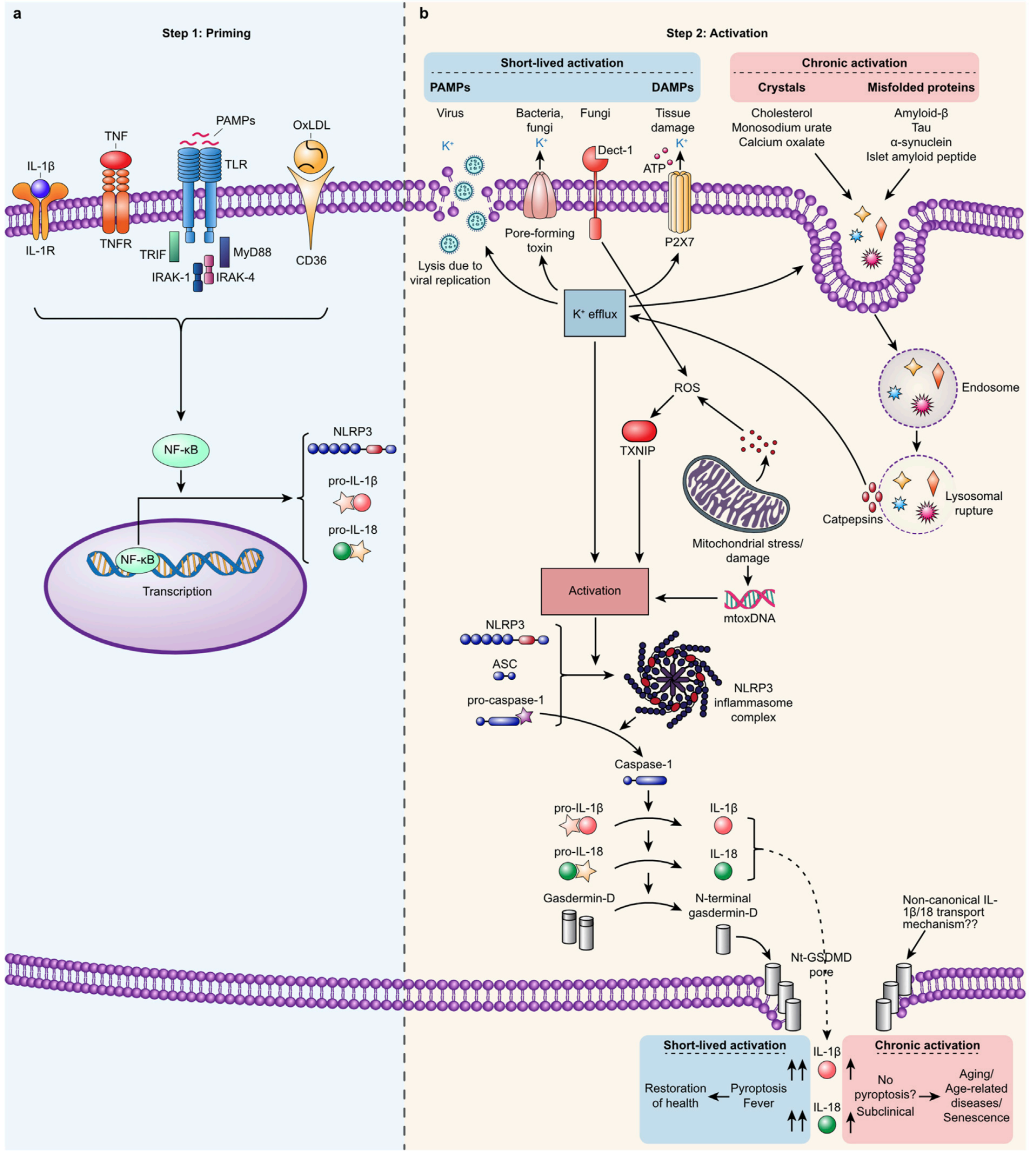
Canonical NLRP3 inflammasome: a two-step process involving priming and activation. **(A)**. Step 1. Priming occurs when extracellular signals such as oxidized LDL, PAMPs like LPS, IL-1α, IL-1β, or TNFs bind to their respective receptors (CD36, TLRs, TNFR, and IL-1R), driving NF-κB-dependent expression of NLRP3, pro-IL-1β, and pro-IL-18. **(B)**. Step 2. A broad spectrum of signals such as PAMPs derived from bacteria, viruses, and fungi and DAMPs, such as extracellular ATP, and mitochondrial stress activate NLRP3 in primed cells. Activation occurs indirectly when K+ efflux, ROS production, or disruption of the cellular membrane is induced or directly by binding mitoDNA, secreted from damaged mitochondrial. Self-derived signals such as cholesterol, monosodium urate, calcium oxalate crystals, and misfolded amyloid-β, Tau, α-synuclein, and islet amyloid peptide can indirectly activate NLRP3. This activation occurs when these substances rupture the lysosomes that contain them, leading to the release of cathepsins. Activated NLRP3 oligomerizes and forms an inflammasome complex that binds the adaptor protein ASC. This complex attracts pro-caspase-1 proteins, which cluster into what becomes the tail of the inflammasome and undergo self-cleavage to form fully active proteases. Active caspase-1 cleaves the substrates pro-IL-1β and pro-IL-18 to form IL-1β and IL- 18, respectively, and cleaves the N-terminal fragment from Gasdermin-D. Once activated, NLRP3 oligomerizes and forms an inflammasome complex that binds to the adaptor protein ASC. This complex then recruits pro-caspase-1 molecules, which cluster and undergo self-cleavage to become fully active proteases. The activated caspase-1 then processes the precursors pro-IL-1β and pro-IL-18 into their active forms, IL-1β and IL-18, respectively. Additionally, activated caspase-1 cleaves the N-terminal fragment from Gasdermin-D, allowing it to assemble into pores in the plasma membrane. These pores facilitate the secretion of active IL-1β and IL-18. Pyroptosis is induced when the concentration of these pores surpasses a certain threshold and the activity of Gasdermin-D pores is prolonged, leading to an inflammatory programmed cell death. In cases of a short and weak response, the number of GSDMD pores remains below the threshold necessary for pyroptosis, allowing cells to secrete low levels of cytokines while staying viable. Typically, DAMPs and PAMPs trigger a robust but short-lived response (lasting hours or days), resulting in pyroptosis and the high-level secretion of IL-1β and IL-18. This leads to clinical symptoms such as fever and facilitates the clearance of the activation signals, ultimately restoring health. Conversely, activation by age- and lifestyle-related signals usually triggers a weaker and more chronic response (lasting months or years), characterized by continuous low-level secretion of IL-1β and IL-18 without clinical symptoms. This prolonged low-grade inflammation contributes to aging, age-related diseases, and cellular senescence. Noncanonical and alternative activation pathways are not depicted in this description.

Resting cells typically exhibit low levels of NLRP3 mRNA, necessitating a priming step to enhance its expression (Bauernfeind et al., 2009). Lipopolysaccharide (LPS) from gram-negative bacteria and double-stranded RNA following viral infections are known PAMPs that upregulate NLRP3 expression. LPS binds to TLR4, while polyriboinosinic:polyribocytidylic acid (PolyI:C), a synthetic double-stranded RNA analog, induces priming via intracellular, endosomal TLR3 stimulation (Chow et al., 1999; Alexopoulou et al., 2001). Additionally, tumor necrosis factor (TNF)-α, a sterile DAMP, primes NLRP3 by binding to the TNF receptor (TNFR). (Franchi et al., 2009). Both TLR4 and TNFR signal through nuclear factor-κB (NF-κB), leading to transcription of not only NLRP3 but also pro-IL-1β, pro-IL-18, and GSDMD (Awad et al., 2017; Muhammad et al., 2019). The mature inflammasome formed after NLRP3 activation cleaves these molecules into their active forms. For a graphical representation, see Figure 1A.

It is noted that NLRP3 mRNA levels are 3–5 times lower in resting compared to LPS-primed human primary peripheral blood mononuclear cells. Notably, the levels of pro-IL-1β are significantly lower in unprimed cells, exhibiting a reduction by several orders of magnitude (Awad et al., 2017). Additionally, GSDMD mRNA is upregulated by a factor of 4–5 following LPS priming (Muhammad et al., 2019). This dramatic difference underscores the profound impact of priming on inflammasome activation, highlighting the importance of regulating NLRP3, Il-1β, and GSDMD mRNA levels in controlling this specific inflammasome-mediated innate immune response. Recent research indicates that NLRP3 priming is a complex process involving not only the upregulation of expression but also transcriptional and post-translational modifications, as well as specific protein interactions, which collectively prime NLRP3 for inflammasome formation (reviewed in (Mckee and Coll, 2020)). The above underscores the crucial role of priming in regulating and activating the NLRP3 inflammasome.

#### 2.1.2 Activation of NLRP3 and inflammasome formation

Various stimuli, including pathogens such as bacteria, viruses, and fungi, and signals indicative of cellular distress activate NLRP3. This broad spectrum of activators indicates that NLRP3 activation extends beyond simple direct receptor interactions, often involving more complex, indirect pathways reflective of cytosolic disturbances. For instance, a critical indirect pathway involves potassium efflux, a phenomenon linked to various stressors, including bacterial toxins and viral replication, significantly contributing to NLRP3 inflammasome activation. This mechanism is well-documented across numerous studies, highlighting its pivotal role in the inflammatory response to cellular stress (Mariathasan et al., 2006; Laudisi et al., 2013; Kasper et al., 2018; Da Costa et al., 2019). Additionally, extracellular adenosine triphosphate (ATP), often released by cells under stress or during cell death, can bind to the P2X7 receptor, prompting potassium efflux and subsequent NLRP3 activation. This process underscores the role of ATP as a “danger signal” in the immune response (Solle et al., 2001). Mitochondrial dysfunction, which produces reactive oxygen species (ROS), is another crucial factor in activating the NLRP3 inflammasome. The ROS generated from impaired mitochondria serve as signaling molecules that can initiate or amplify the inflammatory response by activating the NLRP3 inflammasome (Zhou et al., 2011) Moreover, mitochondrial DNA (mtDNA) has been identified as one of the few molecular patterns capable of directly binding to and activating the NLRP3 inflammasome. The release of mtDNA into the cytosol, often due to mitochondrial stress or damage, can trigger an innate immune response through NLRP3 inflammasome activation (Shimada et al., 2012). Figure 1B provides an overview of these triggers.

Upon activation, NLRP3 transforms and oligomerizes with other activated NLRP3 proteins to form large, prion-like structures. These oligomers attract the apoptosis-associated speck-like protein containing a caspase-recruitment domain (ASC) (Bryan et al., 2009). This assembly then recruits pro-caspase-1, leading to its dimerization. Consequently, caspase-1 cleaves itself to generate a fully active protease (Black et al., 1989; Martinon et al., 2002). Activated caspase-1 then processes pro-IL-1β and pro-IL-18 into their active forms and cleaves GSDMD to release Nt-GSDMD, which form pores in the plasma membrane (Mariathasan et al., 2006; Shi et al., 2015). This event allows the release of active IL-1β and IL-18 and leads to an increase in osmotic pressure inside the cells, causing them to swell and burst, resulting in pyroptosis—an inflammatory form of programmed cell death. Typically, the systemic presence of DAMPs or PAMPs can evoke robust immune responses, such as high fever from the secretion of IL-1β, potentially leading to a hyperinflammatory state that can be detrimental (Ong et al., 2017). Conversely, localized activation tends to induce a more subclinical or mild response. In both scenarios, the response abates once the activators and damaged cells are cleared. Figure 1B illustrates the cascade through which NLRP3 is activated by PAMPs and DAMPs, leading to both robust and mild immune responses.

Traditionally, NLRP3 expression and activation were thought to be confined to cells of the innate immune system, such as activated monocytes, dendritic cells, and macrophages, including specialized types like Kupffer cells and microglia (Guarda et al., 2011). However, it is now understood that NLRP3 is expressed and activated across a broad range of cell types, including epithelial cells, endothelial cells, fibroblasts, hepatocytes, adipocytes, cardiomyocytes, skeletal muscle cells, neutrophils, platelets, T-helper cells, cytotoxic T cells, hematopoietic and mesenchymal stem cells, keratinocytes, and neurons (Vandanmagsar et al., 2011; Thinwa et al., 2014; Xia et al., 2014; Boaru et al., 2015; Arbore et al., 2016; Karmakar et al., 2016; Yao et al., 2017; Boursereau et al., 2018; Chen G. et al., 2018; Sand et al., 2018; Von Herrmann et al., 2018; Cornelius et al., 2019; Ershaid et al., 2019; Ahn et al., 2020; Ratajczak et al., 2020; Javaid et al., 2023). While this ubiquity is advantageous for acute responses, it may contribute to adverse effects in chronic conditions and aging.

### 2.2 Chronic NLRP3 priming and activation drives aging-associated pathologies

Emerging evidence underscores the dual role of NLRP3 inflammasome priming and activation in the progression of age-related diseases. As individuals age, there is an observable increase in both the priming and activation of NLRP3, each contributing uniquely to the inflammatory milieu characteristic of aging. This section delves into the intricacies of these processes, elucidating how age-associated increases in NLRP3 priming and subsequent activation are pivotal in the pathogenesis of various age-related conditions.

#### 2.2.1 An age-related sensitization of NLRP3 expression amplifies low-grade inflammation

Age-related declines in homeostatic mechanisms and dietary accumulations contribute to the increased presence of endogenous components, serving as priming signals for NLRP3. These signals upregulate NLRP3 expression, predominantly through TLR activation, setting the stage for heightened inflammatory responses. Oxidized low-density lipoprotein (oxLDL) immune complexes, for example, enhance the expression of inflammasome-related genes such as IL1B and NLRP3 via CD36 and TLR4 pathways, illustrating the complex interaction between lipid metabolism and inflammation (Rhoads et al., 2017). Similarly, elevated levels of saturated fatty acids (SFAs) are known to prime NLRP3 through TLR4, indicating a dietary link to inflammasome sensitization (Finucane et al., 2015). The pro-inflammatory cytokine TNF-α, whose levels increase with age, contributes to transient and sustained NLRP3 priming, further underscoring the role of chronic inflammation in aging (Franchi et al., 2009). Experimental evidence supports this age-related sensitization: old mice exhibit heightened sensitivity to NLRP3 activators compared to their younger counterparts. Isoflurane, for example, triggers NLRP3 activation leading to cognitive impairment and hippocampal inflammation in aged mice, a response absent in the young (Wang et al., 2018). Similarly, inhalation of the potent NLRP3 activator bleomycin results in lethal outcomes in old WT mice but not in young WT or old NLRP3^−/−^ mice, highlighting the lethal impact of NLRP3 activation in the aged (Stout-Delgado et al., 2016). Furthermore, fibrillar amyloid-β (Aβ) induces IL-1β secretion in microglia isolated from aged, but not young, mice, demonstrating the age-specific activation of NLRP3 by Aβ (Wu et al., 2013). Thus, an increase in NLRP3 priming occurs during aging, which can magnify the response to activation signals and elucidate the heightened vulnerability of older mammals to NLRP3-driven inflammation.

#### 2.2.2 Autologous and dietary sterile NLRP3 activators: Implications for age-related pathologies

Aging is associated with increased self-derived or dietary components, such as misfolded proteins and various types of crystals, that can activate the NLRP3 inflammasome (Hornung et al., 2008). This process leads to chronic, sterile inflammation, which, over years or even decades, contributes to a range of age-related diseases by perpetuating a state of low-grade inflammation (Youm et al., 2013). Figure 1B visually summarizes some of the critical components and their mechanisms of action.

##### 2.2.2.1 Cardiovascular disease (CVD)

The role of NLRP3 in CVD is not to be underestimated. Cholesterol crystals, triggered by metabolic imbalance and high intake of certain fats, activate NLRP3 in peripheral blood mononuclear cells (PBMNCs), a process crucial in atherosclerosis development (Duewell et al., 2010). Cells derived from atherosclerotic plaques show elevated NLRP3 and IL-1β mRNA levels, releasing IL-1β when exposed to LPS and ATP or cholesterol crystals (Paramel Varghese et al., 2016). In animal models, NLRP3 deletion has proven to be a significant preventive measure against atherosclerosis and lesion development (Duewell et al., 2010; Van Der Heijden et al., 2017). NLRP3 activation is also associated with human hypertension and related vascular dysfunction (Dalekos et al., 1997; Grimsgaard et al., 1999; Wen et al., 2011). Experimental interventions silencing NLRP3 in hypertensive rats have shown reduced vascular complications and lower blood pressure, with similar results seen when NLRP3 inhibitors like MCC950 are used (Sun et al., 2017; Krishnan et al., 2019). NLRP3 also plays a role in ischemia-reperfusion injury, affecting outcomes such as infarct and stroke sizes; MCC950 administration in models of arterial occlusion has led to reduced tissue damage (Sandanger et al., 2013; Van Hout et al., 2017; Ismael et al., 2018). Colchicine, a recognized NLRP3 inhibitor traditionally used for gout, has shown the potential to reduce cardiovascular risks in clinical trials, emphasizing NLRP3’s significant role in cardiovascular disease progression and mitigation (Martinez et al., 2015; Nidorf et al., 2020).

##### 2.2.2.2 Obesity-induced insulin resistance and type II diabetes mellitus (DMII)

Aging is intricately linked with an increase in abdominal fat, which not only leads to a heightened release of fatty acids and hormones but also results in an accumulation of adipose tissue macrophages (ATM), fundamentally altering the fat tissue’s physiology (Weisberg et al., 2003). These obesity-induced changes are critical in developing insulin resistance, characterized by a diminished ability of insulin to suppress hepatic glucose production, particularly gluconeogenesis. This inefficiency elevates blood glucose levels, increasing insulin production from pancreatic β-cells. Over time, this compensatory hyperinsulinemia may progress to DMII as β-cells fail to meet the increased insulin demand. At a molecular level, compounds like ceramide, misfolded islet amyloid polypeptides (IAPP), and palmitic acid—significantly increased in altered abdominal fat—are potent activators of the NLRP3 inflammasome, enhancing IL-1β secretion (Ebbesson et al., 2010; Masters et al., 2010; Vandanmagsar et al., 2011; Błachnio-Zabielska et al., 2012). Also, advanced glycation end products (AGEs), resulting from a non-enzymatic reaction between sugars (such as glucose) and proteins or fats, and whose formation can be accelerated by factors such as high blood sugar levels and oxidative stress, can activate NLRP3 (Kong et al., 2017). The impact of NLRP3 inflammasome activation in obesity-induced insulin resistance and DMII is profound. Chronic exposure to IL-1β impairs the ability of human pancreatic islet cells to release insulin in response to glucose, exacerbating glucotoxicity (Maedler et al., 2002). Elevated IL-1β levels are associated with a 3.3-fold increased risk of developing DMII, highlighting its significant role in the disease’s pathogenesis (Spranger et al., 2003). Research also indicates that untreated DMII patients have higher levels of NLRP3 protein in their PBMNC-derived macrophages, which secrete increased levels of IL-1β upon activation (Lee et al., 2013). In experimental models, mice on a high-fat diet develop DMII, but deletion of the NLRP3 gene protects against insulin resistance, pancreatic islet fibrosis, and β-cell death (Vandanmagsar et al., 2011; Youm et al., 2011). Additionally, administering tranilast, an NLRP3 inhibitor, prevents the elevation of fasting glucose levels in these models (Huang et al., 2018). Oral N-acetylcysteine, which acts upstream of NLRP3, ameliorated AGEs-induced pancreatic islet damage and IL-1β formation in mice, similar to the effects observed in NLRP3 knockout models (Kong et al., 2017). Interestingly, NT-0249, a brain-penetrant NLRP3 inhibitor, induced weight loss in obese mice comparable to that caused by semaglutide, an anti-diabetic drug that is also used to treat obesity (Thornton et al., 2024). These findings collectively underscore the crucial role of NLRP3 inflammasome activation in the pathogenesis of insulin resistance and DMII.

##### 2.2.2.3 Cognitive decline and neurodegenerative diseases

Brain aging involves physiological changes such as increased region-specific astrogliosis, marked by alterations in astrocytes’ gene expression, quantity, and structure. These changes, associated with cognitive decline, are prevalent in aging humans and do not necessarily coincide with Aβ or Tau pathology (Bronzuoli et al., 2019; Mohamed et al., 2022). Research in mice indicates that hippocampal astrogliosis, critical for memory, can occur without apparent Aβ or Tau pathology and is exacerbated by a high-fat diet (HFD). Notably, NLRP3 knockout prevented age-related and diet-accelerated astrogliosis and cognitive decline, illustrating NLRP3’s significant role in these processes (Youm et al., 2013). Supporting evidence is that a novel brain-penetrant NLRP3 inhibitor also reverses astrogliosis induced by a high-fat diet (Thornton et al., 2024). Extensive reviews and studies highlight NLRP3’s crucial role in developing neurodegenerative diseases (Heneka et al., 2018). For example, *postmortem* analyses have revealed increased levels of IL-1β and IL-6 in the hippocampi of aged humans, supporting the inflammatory hypothesis of early Alzheimer’s disease (AD) pathogenesis (Cribbs et al., 2012). Elevated IL-1β levels in the cerebrospinal fluid and peripheral blood are linked to AD and Parkinson’s disease (PD) (Blum-Degen et al., 1995; Ng et al., 2018; Fan et al., 2020). Furthermore, NLRP3 activation by misfolded proteins, such as oligomeric Aβ and aggregated Tau in AD and α-synuclein in PD, underscores the inflammatory component of these diseases (Halle et al., 2008; Codolo et al., 2013; Stancu et al., 2019). Genetic and pharmacological inhibition of NLRP3 in various mouse models shows protection against Aβ plaque formation, Tau hyperphosphorylation, rotenone-induced PD, and related cognitive declines, highlighting NLRP3’s potential as a therapeutic target (Heneka et al., 2013; Dempsey et al., 2017; Martinez et al., 2017; Ising et al., 2019). Overall, these findings emphasize NLRP3’s central involvement in age-related astrogliosis and the progression of neurodegenerative diseases.

##### 2.2.2.4 Inflammatory and degenerative joint diseases

Age is the primary risk factor for developing osteoarthritis (OA) and adult-onset rheumatoid arthritis (RA), with aging leading to the deposition of basic calcium phosphate crystals, such as hydroxyapatite, around joints. This deposition contributes to cartilage calcification and OA progression (Dahaghin et al., 2005; Crowson et al., 2011). These crystals activate the NLRP3 inflammasome, producing IL-1β secretion that exacerbates joint damage. Studies indicate that NLRP3 knockout mice exhibit reduced joint pathology, and NLRP3 inhibitors like MCC950 and colchicine have shown protective effects against cartilage degradation and reduced the need for joint replacements (Jin et al., 2011; Pazar et al., 2011; Ni et al., 2021; Heijman et al., 2023). While often considered a lifestyle-related disease, gout exemplifies NLRP3 activation through monosodium urate (MSU) crystal formation due to purine metabolism dysfunction (Martinon et al., 2006). These crystals trigger the NLRP3 inflammasome, increasing IL-1β release from patients’ PBMCs. Colchicine treatment effectively reduces pain during gout attacks, underscoring its role in managing the condition (Terkeltaub et al., 2010; Mylona et al., 2012). In RA, studies have shown that patients’ blood cells release more IL-1β when activated (Choulaki et al., 2015). Targeting the NLRP3 inflammasome, either through genetic manipulation such as the removal of the A20/TNFAIP3 gene, or using NLRP3 inhibitors like MCC905, significantly lessens joint inflammation and damage in mice, illustrating NLRP3’s critical role in RA (Vande Walle et al., 2014; Guo et al., 2018). Corroborating all this, treatment with an anti-IL-1β antibody in patients with atherosclerosis was associated with significant reductions in reported cases of arthritis, gout and osteoarthritis, compared to those receiving a placebo (Ridker et al., 2017b). Collectively, these findings underscore the pivotal role of NLRP3 in the pathogenesis of inflammatory and degenerative joint diseases.

##### 2.2.2.5 Cancer

As the incidence of cancer increases with age, the majority of malignancies are recognized as age-related diseases (Nih, 2021). The NLRP3 inflammasome plays a central role in the pathogenesis of several cancers. Evidence shows that its inhibition or neutralization of IL-1β significantly impacts carcinogenesis and cancer progression. Research involving NLRP3 knockout mice has demonstrated reduced carcinoma incidence, suggesting that NLRP3 activity is linked to cancer development (Marín-Aguilar et al., 2020). For instance, NLRP3 deficiency in methylcholanthrene (MCA)-induced fibrosarcoma models delayed tumor onset and improved tumor-free survival (Chow et al., 2012). Similar protective effects are observed in breast cancer and melanoma models, where NLRP3 knockout decreased tumor growth, reduced metastasis, and enhanced survival (Van Deventer et al., 2010; Guo et al., 2016). Moreover, the potential of NLRP3 inhibitors such as MCC950 and colchicine in suppressing tumor growth has been demonstrated in experimental models of head and neck squamous cell carcinoma and gastric cancer (Chen L. et al., 2018; Zhang T. et al., 2019). The anti-inflammatory effects of IL-1β knockout have also been demonstrated in breast cancer models, where tumor regression was observed (Kaplanov et al., 2019). In clinical settings, chronic use of colchicine has been associated with decreased cancer incidence (Kuo et al., 2015; Lin et al., 2023). Notably, the clinical use of canakinumab, an anti-IL-1β agent, has shown significant promise, as it reduced the incidence of lung cancer in patients with atherosclerosis, providing a robust clinical validation of the critical role of NLRP3 and IL-1β in cancer pathophysiology (Ridker et al., 2017b).

## 3 The molecular interplay between NLRP3, aging signaling pathways, and cellular senescence

**FIGURE 2:**
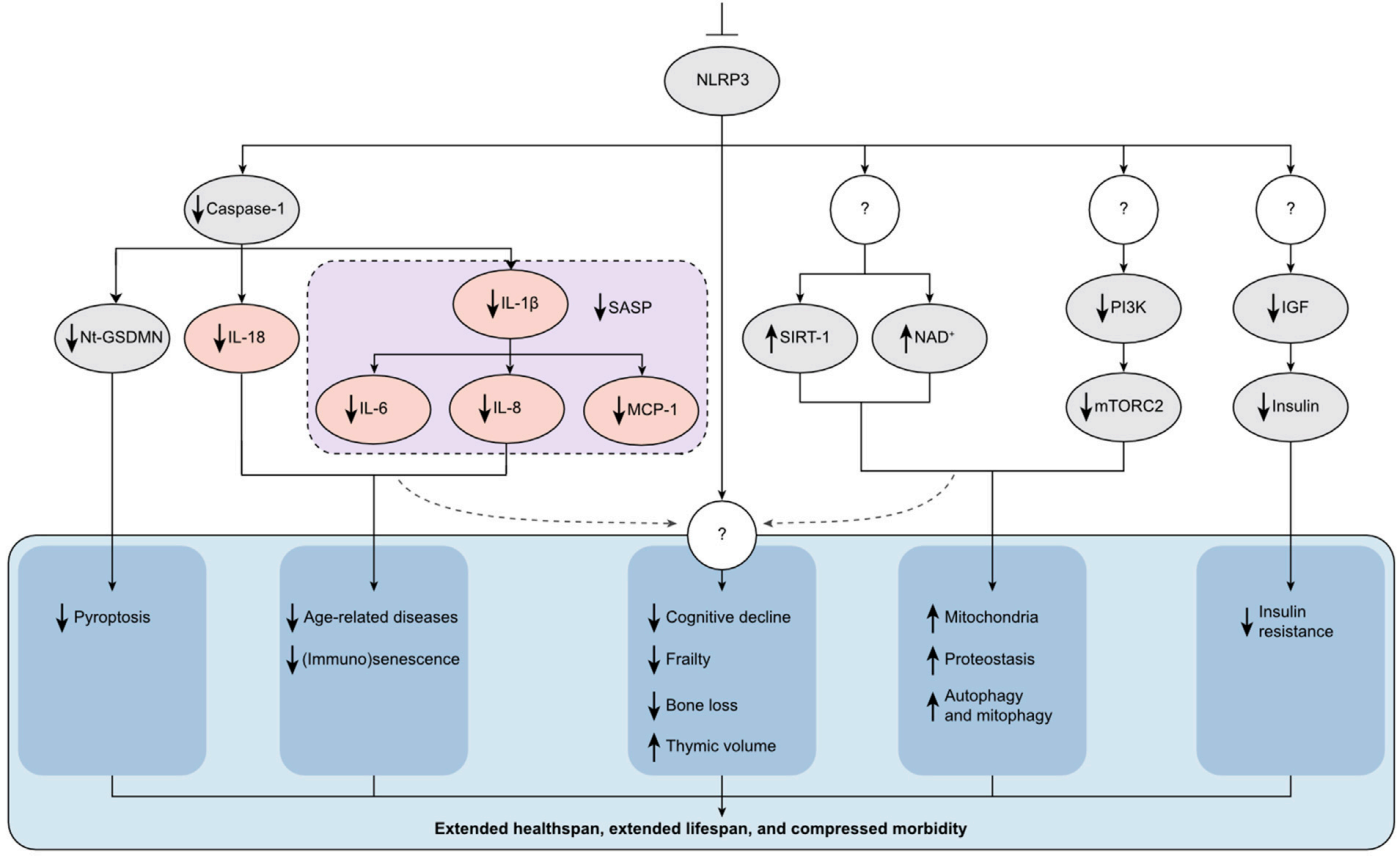
NLRP3’s Role in Aging and Senescence. NLRP3 operates upstream of numerous molecular pathways associated with aging and cellular senescence, presenting a viable target to extend healthspan (HS) and lifespan (LS). Inhibition of NLRP3 through genetic deletion or the use of specific small-molecule inhibitors reduces the production of inflammatory cytokines such as IL-1β, IL-18, IL-6, and TNF-α (illustrated in pink ovals). These cytokines are key contributors to the pathology of age-related diseases and are also part of the senescence-associated secretory phenotype (SASP) secreted by senescent cells. Additionally, blocking NLRP3 mitigates immunosenescence. Furthermore, inhibition of NLRP3 positively influences several metabolic pathways critical to aging. For instance, it elevates levels of SIRT-1 and NAD+, decreases mTORC2 activity, and reduces IGF and insulin levels. Consequently, symptoms of aging such as cognitive decline, frailty, bone loss, thymic involution, and insulin resistance are delayed. This comprehensive modulation of aging pathways results in extended HS and LS, and a reduction in morbidity. The figure also includes question marks to denote steps and mechanisms that remain unknown or not well understood, highlighting areas for future research.

Central to aging are pathways such as autophagy, which is crucial for removing intracellular debris; mitochondrial homeostasis, which is essential for managing energy and ROS; and hormonal signaling, which is vital for growth and tissue health. Aging also involves DNA damage, telomere shortening, and epigenetic alterations, which undermine genomic integrity and accelerate the decline in health. These processes typically culminate in cellular senescence, marked by halted cell division, functional loss, and the secretion of inflammatory signals, all contributing to stem cell exhaustion and exacerbating aging. Notably, a myriad of aging mechanisms, including inflammaging (driven by cellular debris and misfolded proteins), metaflammation (fueled by excessive nutrient intake, dietary patterns, and gut microbiota dynamics), and garbaging (the failure to clear damaged cellular materials), appear to converge on NLRP3-mediated low-grade inflammation. This suggests a unifying inflammatory basis for aging processes, as proposed by Franceschi et al. (Franceschi et al., 2018), highlighting the profound link between metabolism, inflammation, and aging. However, the intricate relationship between NLRP3 and aging pathways is not easily deciphered. NLRP3 often acts upstream in key aging-related processes, indicating a complex and bidirectional interplay. This intricate relationship underscores NLRP3’s dual role in aging and senescence, which will be further explored in this chapter. Figure 2 provides a visual representation of how NLRP3 influences various molecular pathways involved in aging.

### 3.1 NLRP3 interactions with key aging mechanisms

Autophagy, a critical cellular clearance mechanism, declines with age, significantly contributing to aging. The mammalian target of rapamycin (mTOR) and AMP-activated protein kinase (AMPK) are two key regulators of autophagy. For instance, mTOR inhibition by rapamycin enhances autophagy, extending HS and LS in mice (Harrison et al., 2009). Autophagy activation also reduces IL-1β production by moderating NLRP3 activation and lowering pro-IL-1β levels (Harris et al., 2011). Conversely, NLRP3 regulates autophagy; its knockout prevents the age-associated decline in autophagy typically seen in older, healthy mice (Marín-Aguilar et al., 2020; Pavillard et al., 2017). Additionally, inhibition of NLRP3 with MCC950 helps forestall the decline in autophagy associated with aging (Pavillard et al., 2017; Marin-Aguilar et al., 2020). AMPK, activated by increased AMP:ATP ratios indicative of ATP consumption, enhances autophagy and mitochondrial function. Caloric restriction (CR), known for extending LS, activates AMPK, underscoring its crucial role in longevity (Slack et al., 2012). However, inhibiting AMPK activates NLRP3 and increases IL-1β levels (Bullon et al., 2016).

Metformin, a diabetes drug under investigation for its potential to extend HS and LS, activates AMPK and inhibits the NLRP3 inflammasome (Zhou et al., 2001; Barzilai et al., 2016; Yang et al., 2019). Similarly, the HS and LS-extending effects observed with CR and metformin treatment mimic those seen in NLRP3 knockout mice, highlighting NLRP3’s pivotal role as a critical regulator in these longevity-enhancing pathways (Bullon et al., 2016; Marín-Aguilar et al., 2020). Moreover, MCC-950 treatment in obese mice increased AMPK phosphorylation levels, further illustrating the intertwined roles of AMPK activation and NLRP3 inhibition in aging (Wang et al., 2022).

Diminished mitochondrial function is a critical contributor to aging. Mitochondrial metabolic responses to diet and exercise are regulated by sirtuins (SIRT1-7), which depend on the coenzyme Nicotinamide adenine dinucleotide (NAD)+. SIRT1 is vital in maintaining cellular homeostasis in various tissues, including the liver, skeletal muscle, adipose tissue, and brain. A decline in NAD+ levels is directly linked to mitochondrial dysfunction. This condition is exacerbated by the deletion of SIRT1, leading to increased production of ROS and subsequent activation of NLRP3 (Zhou et al., 2011; Gomes et al., 2013). Moreover, a decrease in intracellular NAD+ has been associated with NLRP3 activation, whereas NAD+ supplementation has been shown to reduce IL-1β production (Misawa et al., 2013). Mitochondrial dysfunction is a well-documented driver of age-related diseases, which can be mitigated by enhancing mitochondrial function. For instance, elevating SIRT1 expression specifically in the brain has been shown to extend LS in mice (Balaban et al., 2005; Satoh et al., 2013). While whole-body overexpression of Sirt1 improved HS without affecting LS, the use of SRT1720, a selective SIRT1 activator, extended both HS and LS in mice on a standard diet (Herranz et al., 2010; Mitchell et al., 2014). Similarly, nicotinamide mononucleotide (NMN), a precursor of NAD+, boosted mitochondrial oxidative metabolism and enhanced HS in mice (Mills et al., 2016), whereas nicotinamide (NAM) and nicotinamide riboside (NR), other NAD+ precursors, showed varied effects on HS and LS (Mitchell et al., 2018; Harrison et al., 2021). Intriguingly, NLRP3 knockout in aged mice resulted in a significant increase in NAD+, SIRT1, and mitochondrial levels, indicating that NLRP3 acts upstream of processes crucial for mitochondrial function (Marín-Aguilar et al., 2020).

The insulin-like growth factor-1 (IGF-1) pathway is a critical regulator of survival, proliferation, metabolic homeostasis, HS, and LS. For example, CR induces lower levels of insulin and IGF-1, which correlate with improved HS and increased longevity (Breese et al., 1991). Notably, humans exhibiting exceptional longevity have reduced IGF-1 levels, suggesting a link between lower IGF-1 and extended LS (Milman et al., 2014).

The growth hormone (GH)/IGF-1 axis is another pivotal regulator of metabolism and aging. For instance, Ames dwarf mice, which have impaired GH production, exhibit significantly extended LS’s compared to their WT counterparts, while mice with elevated GH levels have reduced LS’s, inversely proportional to their GH plasma levels (Pendergrass et al., 1993; Brown-Borg et al., 1996). GH appears to regulate IGF-1 and NLRP3, as evidenced by GH receptor-knockout (GHRKO) mice. These mice have low serum IGF-1 levels, diminished age-related NLRP3 inflammasome activation in response to NLRP3 ligands, and a 38%–55% longer LS than WT mice (Coschigano et al., 2000; Spadaro et al., 2016). Furthermore, blocking the IGF-1 receptor in aged mice with monoclonal antibodies resulted in a female-specific increase in both HS and LS (Mao et al., 2018). Interestingly, NLRP3 has been shown to regulate IGF-1 levels, as both young and old NLRP3^−/−^ mice exhibit significantly lower IGF-1 levels, a finding further substantiated by reduced serum IGF-1 levels in aged mice treated with the NLRP3 inhibitor MCC950 (Marin-Aguilar et al., 2020; Marín-Aguilar et al., 2020). This positions NLRP3 as an upstream regulator of the IGF-1 signaling pathway. A corroborating finding is that NLRP3 ablation improves glycemic control in aged mice on a standard diet, further highlighting the complex interplay between NLRP3, the GH/IGF-1 axis, and metabolic homeostasis in the context of aging (Youm et al., 2013).

Epigenetic alterations, including DNA methylation, histone modification, and microRNA signaling, are vital mechanisms regulating gene expression by modifying DNA accessibility and chromatin structure without altering the DNA sequence. These changes profoundly influence gene activity and cellular phenotype throughout an organism’s LS. For instance, SIRT1 deficiency leads to hypomethylation at CpG sites on the IL-1β proximal promoter, activating its transcription (Cho et al., 2015). Additionally, IL-1β triggers demethylation at specific sites on the promoters of pro-inflammatory genes IL6 and IL8, increasing their expression and secretion from human intestinal epithelial cells (Caradonna et al., 2018). Furthermore, microRNA-223 (miR-223) overexpression decreases NLRP3 expression and subsequent IL-1β secretion in primed and activated macrophages (Bauernfeind et al., 2012). These examples highlight the complex interplay of regulatory mechanisms in gene regulation.

Telomeres, consisting of tandem repeats at chromosome ends, safeguard against progressive degradation and prevent chromosomal fusion. With each somatic cell division, telomeres naturally shorten, necessitating telomerase activity to maintain their length. Inadequate maintenance leads to telomere dysfunction, which primes for NLRP3 expression and subsequent hyperactivation upon activation (Kang et al., 2018). The role of telomeres extends to aging; their attrition contributes to DNA damage, replicative senescence, and age-related diseases and is inversely correlated with LS (Samper et al., 2001; Artandi and Attardi, 2005). Counteracting this process, telomere extension mitigates DNA damage and senescence, enhances cognitive function, and promotes both HS and LS in mice (Bernardes De Jesus et al., 2012; Muñoz-Lorente et al., 2019). Interestingly, NLRP3 appears to regulate telomerase activity, as evidenced by NLRP3 knockout mice exhibiting elongated telomeres *in vivo* (Marín-Aguilar et al., 2020), suggesting a role for NLRP3 in the intricate balance between telomere maintenance, cellular aging, and longevity.

Thus, NLRP3 plays a central and multifaceted role in aging through its complex interactions with critical biological processes, including autophagy, mitochondrial function, the GH/IGF-1 axis, epigenetic regulation, and telomere integrity.

### 3.2 NLRP3 interactions with low-grade inflammation and senescence

As previously discussed, aging is associated with an increase in self-derived and dietary signals, oxidative stress from mitochondrial dysfunction, and elevated cytokines, all of which can prime or activate NLRP3. Concurrently, aging is marked by a subtle rise in inflammatory markers such as IL-6 and C-reactive protein (CRP), associated with frailty and increased mortality in the elderly (Parrinello et al., 2015; Puzianowska-Kuznicka et al., 2016). While IL-1β levels do not consistently increase with age, blocking IL-1β with antibodies reduces IL-6 and CRP levels, confirming IL-1β′s role upstream of these markers (Ridker et al., 2017a). This observation suggests that NLRP3-mediated IL-1β secretion, tightly regulated due to its potent pyrogenic effects, leads to minimal local production in response to the relatively low amount of sterile, age-related activators in healthy individuals. Consequently, systemic levels of IL-1β may stay near or below detection limits. However, its effect is amplified by the downstream production of IL-6 and CRP, which are easily detectable and thus serve as sentinels or surrogate markers for NLRP3-mediated low-grade inflammation. A meta-analysis of clinical trials with coronary artery patients revealed that colchicine intervention was associated with reducted IL-6 and hs-CRP levels (Pan et al., 2022). Additionally, a placebo-controlled trial involving the oral administration of a high dose of GlyNAC—a combination of glycine (Gly) and n-acetyl-L-cysteine (NAC), which increases intracellular glutathione and reduces oxidative stress—showed significant reductions in IL-6 and hs-CRP levels, alongside increased insulin sensitivity, gait speed, and muscle strength in healthy individuals over age 60 (Kumar et al., 2023). Others have found that intracellular glutathione acts as a negative regulator of the NLRP3 inflammasome (Zhang et al., 2021). This discovery provides a surprising link to the complex interplay of aging mechanisms through NLRP3.

Intriguingly, the characteristics of low-grade inflammation significantly overlap with those of age-related senescence. This overlap is evident when considering the senescence-associated secretory phenotype (SASP), characterized by the secretion of pro-inflammatory cytokines and chemokines such as IL-1α, IL-1β, IL-6, IL-8, and MCP-1. Senescent cells, often called ‘zombie cells,’ play a dual role in health and aging. While they can contribute significantly to tumor suppression, during aging these cells secrete compounds that disrupt normal organ and tissue functions. This activity propels age-related pathologies and contributes to a decline in overall health, potentially shortening HS (Baker et al., 2011). Both IL-1α and IL-1β are essential for inducing SASP, as inhibiting the binding of either cytokine to the IL-1 receptor (IL-1R) blocks SASP development (Lau et al., 2019). Since active caspase-1 is necessary to produce IL-1β, and its inhibition reduces SASP, coupled with the fact that senescent cells secrete IL-1β, it suggests that the NLRP3 inflammasome is a likely driver of SASP development (Acosta et al., 2013; Latz and Duewell, 2018). Further evidence of NLRP3’s role comes from studies showing its overexpression exacerbates the senescent phenotype in inherently senescent primary mesenchymal stromal cells from patients with myelodysplastic syndrome by increasing IL-1β secretion (Shi et al., 2019). Compellingly, treatment with IL-1β alone is sufficient to induce senescence in human umbilical vein endothelial cells (HUVECs), vascular smooth muscle cells (VSMCs), and mesenchymal stromal cells (Yin et al., 2017; Shang et al., 2020). Moreover, IL-1β has been identified as the sole cytokine capable of driving cellular aging by inducing senescence (Han et al., 2020). This senescence-inducing effect of IL-1β is counteracted by the NLRP3 inhibitor MCC950, which has shown efficacy in preventing senescence in chondrocytes and endothelial cells, highlighting potential therapeutic avenues to mitigate senescence (Ni et al., 2021; Romero et al., 2022). Targeted NLRP3 inhibition, whether through short hairpin RNA or MCC950 treatment, has shown promise in reducing radiation-induced and IL-1β-induced senescence in diverse cell types, including U87 human glioblastoma cells and HUVECs (Li and Liu, 2015; Romero et al., 2022). In another study, colchicine prevented ethanol-induced senescence in human endothelial cells (Zhou et al., 2023).

The role of NLRP3 in immunosenescence, which decreases immune system effectiveness and raises susceptibility to infections in the elderly, is particularly noteworthy. Aged NLRP3^−/−^ mice show significant resistance to age-related thymic decline and support a more diverse T-cell repertoire, indicating a potential protective effect against immunosenescence (Youm et al., 2012). Given NLRP3’s established role in promoting senescence and SASP, the emerging concept that NLRP3 activation does not invariably lead to pyroptosis warrants further exploration. This notion is pivotal as senescent cells, which secrete NLRP3-mediated SASP components, can remain viable for extended periods *in vitro* (De Cecco et al., 2013; Kim et al., 2013; Yin et al., 2017). Such longevity would be implausible if pyroptosis were an inevitable outcome of NLRP3 activation in every senescent cell, suggesting that alternative pathways might be operational. Interestingly, the nature of the activating signals influences NLRP3 inflammasome outcomes significantly. Sterile signals generate weaker and more delayed macrophage responses than microbial signals, leading to lower and prolonged IL-1β secretion and reduced pyroptosis rates. This difference is particularly pronounced in murine cells, where a double sterile signal leads to substantially lower pyroptosis levels than a double microbial signal. However, both types of signals result in comparably low pyroptosis levels in human primary macrophages, indicating species-specific differences in NLRP3-induced pyroptosis kinetics (Bezbradica et al., 2017). Furthermore, a hyperactive cellular state has been identified where NLRP3-dependent IL-1β release can occur without pyroptosis. For instance, the removal of the Sterile alpha and HEAT Armadillo motif-containing protein (SARM) containing a Toll-IL-1R (TIR) domain drives cellular hyperactivation, allowing cultured cells to secrete IL-1β for days while remaining viable (Wolf et al., 2016; Zanoni et al., 2017; Evavold et al., 2018). An alternative pathway involves priming human monocytes with a TLR4 ligand (LPS) without an activation signal. This scenario leads to chronic, low-level NLRP3-mediated IL-1β secretion without inducing cell death, a process that can be inhibited by MCC950 (Gaidt et al., 2016). Notably, IL-1β production through this alternative activation pathway is reduced by approximately one order of magnitude compared to that observed with canonical activation pathways. Moreover, it does not trigger pyroptosis. This suggests that such alternative inflammasome pathways could significantly contribute to age-related low-grade inflammation, wherein IL-1β levels are modestly but chronically elevated. Figure 1B visually represents such non-canonical transport mechanisms for chronic, pyroptosis-free IL-1β secretion.

Thus, NLRP3 plays a pivotal role in orchestrating cellular and immunosenescence, underscoring its crucial significance in aging.

## 4 Targetting NLRP3 to extend HS

Youm et al. were the first to hypothesize that reducing NLRP3 may extend HS by delaying multiple age-related degenerative changes in the periphery and the brain. Their seminal study showed that NLRP3 ablation prevented thymic involution, brain astrogliosis, bone loss, and cognitive decline. Additionally, NLRP3^−/−^ mice showed increased latency to fall on a rotarod and increased running time and distance on a treadmill. Overall, the deletion of NLRP3 extended HS by preventing functional decline in multiple organs (Youm et al., 2013). Subsequent studies further supported the notion that a reduction in NLRP3 could extend both HS and LS. For instance, treating 18-month-old mice with MCC950 for 12 weeks improved their health. Compared to their sham-treated littermates, autophagy, and glucose tolerance were increased, age-related hepatic dysfunction was ameliorated, and fasting blood glucose and circulating IGF-1 levels, as well as body weight, were reduced (Marin-Aguilar et al., 2020). Additional studies confirmed the HS-extending effects of NLRP3 ablation. Older mice typically appeared gray and frail with alopecic fur, whereas knockout mice remained vital with thick, dark, and shiny fur. NLRP3^−/−^ mice also exhibited preserved cardiac integrity and reduced metabolic aging. Remarkably, male and female NLRP3^−/−^ mice showed increases in their mean and maximum LS by 34% and 29%, respectively (Marín-Aguilar et al., 2020). Another study revealed that approximately 70% of twenty-month-old WT mice on a high-fat diet developed ulcerating dermatitis, a severe inflammatory skin disorder, in contrast to only 5% of twenty-month-old NLRP3^−/−^ mice. Moreover, the LS of high-fat diet-fed NLRP3^−/−^ mice was 16% higher than that of their WT counterparts on the same diet and comparable to the LS of WT mice fed a standard diet (Cañadas-Lozano et al., 2020). Further evidence from a progeria mouse model showed that MCC950 treatment to inhibit NLRP3 prevented age-related weight loss and extended mean LS by 19.2% (González-Dominguez et al., 2021). Additionally, bats, known for their extraordinary longevity, exhibit a unique bat-specific splice variant and an evolutionarily adapted LRR domain of NLRP3, thought to dampen the inflammatory response and contribute to their health and longevity by reducing chronic inflammation (Austad and Fischer, 1991; Kacprzyk et al., 2017; Ahn et al., 2019). This observation is supported by findings in long-living naked mole rats, who display a notably dampened immune response, further associating reduced inflammation with extended HS and LS (Oka et al., 2022)

In most humans, an increase in age is accompanied by an elevation in the levels of pro-inflammatory proteins or biomarkers, which are linked to age-related diseases and increased mortality (Cesari et al., 2004). Lower levels of IL-6 are observed in healthy agers—individuals over age 65 who are free from disease, have no disabilities, and maintain good cognition—and seem to predict a prolonged LS. A study of these healthy agers, with a mean age of 76.3 ± 7.9 years, demonstrated that those in the lowest quartile of IL-6 levels exhibited a sevenfold decrease in 5-year mortality rate compared to those in the highest quartile (Puzianowska-Kuznicka et al., 2016). While lower IL-6 levels are linked to extended LS in healthy agers, IL-1β, regulated by the NLRP3 inflammasome, appears to play a crucial upstream role. Research shows that blocking IL-1β reduces IL-6 levels in humans, indicating that IL-1β initiates IL-6 production (Lachmann et al., 2009; Ridker et al., 2017a).

As described in chapter 3, increased NLRP3 priming and activation are closely associated with age-related diseases. However, the role of NLRP3 extends beyond these conditions, influencing the HS preceding the onset of disease. This is supported by findings that activated monocytes from elderly individuals express three times as much NLRP3 as those from younger individuals. Notably, healthy centenarians exhibit NLRP3 levels similar to young people, in contrast to their diseased counterparts, who display levels akin to those of older individuals with health conditions (Tedone et al., 2019). Significantly, a study on 80-year-old master athletes (MA), who engage in rigorous training for approximately 14 h per week, offers a compelling real-life illustration. These MA individuals demonstrate greater muscle mass and superior cardiovascular fitness, muscle strength, and muscle volume compared to their non-athletic (NA) counterparts of the same age. Muscle biopsies from the NA group revealed elevated levels of proteins involved in NLRP3 priming and activation (NF-kB, PYCARD (ASC), and NF-kB), which were significantly lower in the MA group. This suggests that the inflammasome proteins that typically increase with aging are mitigated in physically active elderly subjects (Ubaida-Mohien et al., 2022). Interestingly, the inhibition of NLRP3 seems to play a role in the HS- and LS-extending effects of the ketogenic diet developed to treat therapy-resistant epilepsy. The underlying mechanism for its anti-aging effect is largely unknown. A ketogenic diet, which consists of more than 85% fat and involves the formation of ketone bodies such as β-hydroxybutyrate (BHB) as an alternative energy source for ATP, was shown to extend the HS and LS of mice (Roberts et al., 2017). BHB lowers IL-1β levels by blocking the NLRP3-mediated inflammasome formation, which could explain its positive effect on aging (Youm et al., 2015). In addition to its verified effect in treating epilepsy in children, an increasing body of evidence suggests that a ketogenic diet can extend HS by preventing diseases such as DMII and AD (Goday et al., 2016; Taylor et al., 2018). These observations collectively suggest that NLRP3 expression correlates with the presence of age-related diseases and significantly impacts the HS of aging populations. Despite NLRP3’s central role in aging, a clear correlation between aging and an increase in systemic IL-1β levels has not yet been established. This is likely because IL-1β levels are typically extremely low in human serum. In patients with CAPS, who overproduce IL-1β, serum levels still fall below the detection limit of 0.1 pg/mL for the assays used (Lachmann et al., 2009). In another study, serum IL-1β levels in healthy individuals were found to average 0.2 pg/mL, barely above the detection limit of another ultra-sensitive ELISA assay. This consistency across different age groups led researchers to conclude there was no significant association between age and IL-1β levels (Di Iorio et al., 2003). However, the lack of independent replications, the observation of higher serum IL-1β levels compared to those in CAPS patients, and the discontinuation of the previously used assay all warrant caution in interpreting these results, highlighting the need for more accurate IL-1β detection methods. Moreover, based on estimates that healthy individuals and CAPS patients produce approximately 6 ng and 31 ng of IL-1β per day, serum levels should be up to 500 times higher than typically detected (Lachmann et al., 2009).

One explanation for the extremely low systemic presence of IL-1β is that ELISA assays primarily measure free IL-1β. In serum, IL-1β is neutralized by soluble IL-1 receptors, such as Soluble IL-1 Receptor Types I and II (sIL-1RI and sIL-1RII), which are present at concentrations up to 1000 times higher than those of IL-1β, effectively rendering it biologically inactive. (Symons et al., 1995; Lachmann et al., 2009). Consequently, these soluble receptors act as a buffer, neutralizing the levels of IL-1β that are typically produced in healthy individuals and CAPS patients. Given that the soluble IL-1 receptors effectively neutralize IL-1β levels, resulting in indistinguishable systemic IL-1β concentrations between CAPS patients—who typically overproduce this cytokine—and healthy individuals, it is even less likely to observe differences in IL-1β levels among healthy individuals across different age groups. Therefore, different methods are required to accurately reflect local IL-1β production, such as assays that measure total IL-1β in serum. Alternatively, IL-6 can be used as a sentinel biomarker for local IL-1β production, as suggested by Hoffman et al. (Hoffman et al., 2004). Together, these studies provide compelling evidence that NLRP3 is crucial for healthy aging. However, they also highlight that serum IL-1β, as measured by current methodologies, does not serve as a reliable biomarker for its activation or lack thereof.

The quest for longevity has historically focused on identifying compounds that extend LS in model organisms such as mice. However, translating these findings to humans presents substantial challenges, primarily due to the impracticality of LS studies in humans and the growing realization that extending HS—ensuring longer periods of good health rather than merely prolonging the duration of disease—is fundamentally more important. Therefore, recent scientific efforts have pivoted towards extending HS—the period of life spent in good health—in larger mammals and humans. Given the ethical and logistical constraints of conducting LS-extending studies in healthy humans, this shift is not only pragmatic but also necessary. Current regulations typically restrict the use of experimental drugs to individuals diagnosed with a disease. This regulatory environment complicates interventions aimed at healthy individuals to prolong their HS. As a result, alternative approaches, such as using nutritional supplements, are being explored. In this context, the recently announced XPRIZE Healthspan of €101 million marks a significant incentive (XPRIZE, 2023). The prize will be awarded to the first who can demonstrably reverse specific aging markers—such as declines in muscle strength, cognition, and immune function—by at least a decade. While the precise methodologies, or endpoints, for measuring these reversals are still being refined, the strategy of incrementally reversing aging represents a highly achievable short-term goal. Focusing on reversing specific aging symptoms, rather than extending lifespan, appears to be a pragmatic and attainable objective, which could potentially accelerate developments in the field of health extension.

This section delves into the therapeutic strategies in development that target the NLRP3 inflammasome, either directly or indirectly—first some promising studies in mice that require confirmation in human trials. Rapamycin, which stimulates autophagy by inhibiting mTOR, has been shown to extend the LS of old mice in the rigorous Interventions Testing Program (ITP) (Harrison et al., 2009). Additionally, the autophagy induced by rapamycin inhibits NLRP3 activation and subsequent secretion of IL-1β in a human macrophage cell line (Ko et al., 2017). Treatment with nicotinamide riboside (NR), a variant of vitamin B3, replenishes nicotinamide adenine dinucleotide (NAD+) levels and improves mitochondrial function, thereby prolonging the LS of old mice (Zhang et al., 2016). In a mouse model for cardiac hypertrophy, NR treatment attenuated NLRP3 activation and reduced IL-1β levels (Ma et al., 2021). Nicotinic acid, another form of vitamin B3, enhances NAD+ levels and inhibits NLRP3 activation (Li et al., 2016). Spermidine, a natural polyamine, improves the LS of old mice by promoting autophagy and reducing inflammation (Eisenberg et al., 2016). In a human macrophage cell line, spermidine inhibited NLRP3 inflammasome activation and IL-1β production (Li et al., 2022).

Studies conducted in non-human primates and humans are more directly relevant to human health. For instance, taurine, a semi-essential food ingredient, exhibits significant declines in levels with aging across mice, monkeys, and humans, which is associated with age-related diseases. In aged rhesus macaques (*Macaca mulatta*) supplemented with taurine, notable improvements were observed: increased bone density, reduced fasting blood glucose levels, and lowered serum concentrations of liver damage markers AST and ALT. Additionally, taurine treatment decreased WBCs, monocytes, and granulocytes, which typically increase with age (Singh et al., 2023). In rodents, taurine supplementation has been shown to alleviate age-related diseases by inhibiting NLRP3 activation and reducing IL-1β production (Lee et al., 2021; Qiu et al., 2018).

Metformin, commonly used to manage DMII by reducing glucose production and enhancing insulin sensitivity, offers benefits beyond glycemic control. It is associated with the prevention of CVD, a reduced incidence of cancer, improved cognitive functions, and decreased mortality rates among patients with DMII. Additionally, metformin lowers IGF-1 levels, inhibits mTOR, reduces activity in mitochondrial complex 1 of the electron transport chain, and decreases the endogenous production of ROS. It also activates AMP-activated protein kinase (AMPK), reduces inflammation and may delay senescence (reviewed in (Mohammed et al., 2021). A large clinical trial is underway to establish whether metformin extends the HS of healthy individuals over age 65 (Barzilai et al., 2016). The broad spectrum of metformin’s effects raises the question of whether these benefits are due to multiple mechanisms or the downstream consequences of its primary action on a singular aging-related pathway. Given that metformin also acts as an NLRP3 inhibitor, it is plausible that many, if not all, of these beneficial effects are mediated through this pathway (Zhang L. et al., 2019; Xian et al., 2021).

Age-related declines in the amino acids glycine (Gly) and cysteine (Cys) and the antioxidant glutathione (GSH)—the primary building blocks of which are Gly and Cys—are observed in both mice and humans. These declines are associated with aging and various age-related diseases (Yang et al., 1995; Lyons et al., 2000; Sekhar et al., 2011). GSH, a crucial regulator of mitochondrial health, neutralizes ROS. Its levels can be replenished through the oral administration of Gly and NAC, an acetylated form of Cys known for enhanced oral bioavailability (Samiec et al., 1998; Townsend et al., 2003; Sekhar et al., 2011). In experimental models, Gly alone has been shown to modestly extend the LS of mice in ITP studies (Miller et al., 2019). Furthermore, the oral intake of the combination of Gly and NAC (GlyNAC) has demonstrated a significant 24% increase in the LS of aged mice (Kumar et al., 2022). In healthy individuals over age 70, oral NAC administration significantly improved muscular performance compared to placebo-treated, age-matched controls (Hauer et al., 2003). The administration of GlyNAC to healthy individuals over age 60 significantly improved muscle strength, insulin resistance, and systolic blood pressure. Furthermore, biomarkers of endothelial dysfunction (sVCAM-1), oxidative stress (TBARS), and inflammation (IL-6) showed significant improvements compared to placebo controls (Kumar et al., 2023). In a non-controlled pilot study, GlyNAC significantly improved cognition (Kumar et al., 2021). Traditionally, GSH is known as a ROS scavenger that indirectly modulates NLRP3 activation. However, recent findings reveal that GSH also directly regulates NLRP3 activation. ATP-induced GSH depletion in macrophages triggers NLRP3 inflammasome activation, likely by destabilizing the thioredoxin-interacting protein (TXNIP)-thioredoxin (TRX) complex, thereby freeing TXNIP to activate NLRP3. Furthermore, the exogenous administration of GSH or its oxidized form, GSSG, has been found to reduce IL-1β production and inhibit NLRP3 inflammasome activation significantly (Zhang et al., 2021). These findings not only clarify GSH’s comprehensive role in inflammatory regulation but also illuminate its potential as a promising therapeutic target for managing NLRP3-mediated conditions. Further supporting the significance of glutathione in aging, research indicates that HC exhibit higher GSH:GSSG (GSH disulfide) ratios and total GSH levels compared to individuals aged 70-79. This suggests that elevated glutathione levels may protect against oxidative stress and NLRP3 activation, contributing to these individuals’ exceptional HS and LS (Paolisso et al., 1998; Alonso-Fernández et al., 2008).

Thus, NLRP3 plays a critical role in modulating both HS and LS. Given that self-derived signals primarily drive it, the term ‘auto-aging’ is proposed to describe the accelerated aging mediated by NLRP3 in the broader population. As research progresses, the potential of targeting this inflammasome to mitigate age-related diseases and significantly enhance the quality of life during aging becomes increasingly promising.

## 5 Conclusion and future directions

Given that HC achieve an exceptional HS of at least one hundred years and considering the mounting evidence of their genetic advantages that protect them from age-related diseases, this review makes clear that the vast majority of the population experiences of form of accelerated aging. With the average HS of the broader population falling short by approximately 30 years, a pivotal challenge emerges: identifying these beneficial genetic traits and determining which can be targeted to enhance the HS of the general public. While the aging process remains complex, emerging research continues to illuminate the critical mechanisms involved. NLRP3, a protein primarily known for its role in innate immunity and its significant contributions to age-related diseases, is central to this phenomenon of accelerated aging. However, this does not exclude the involvement of other mechanisms that have a central role in aging. Recently, IL-11 was found to play a central role in mouse aging, with blocking IL-11 extending the LS and HS of aged mice by 24.9% (Widjaja et al., 2024). Today, the biological function of IL-11 remains poorly understood and its role has long been confusing. Recombinant human (rh) IL-11 has shown protective effects against age-related diseases in mice, whereas recombinant murine IL-11 causes age-related diseases (reviewed in Cook, 2023). Cook’s review also shows that there is no evidence that rhIL-11 harms human HS, as it remains an effective treatment for thrombocytopenia. Additionally, clinical studies with rhIL-11 antibodies in humans have not shown favorable outcomes on age-related diseases. Therefore, the role of IL-11 in human aging needs further investigation to determine if the effects observed in mice are species-specific.

This paper presents a novel perspective, amassing substantial evidence that targeting NLRP3 not only converges various aging mechanisms but also exerts regulatory control over them. This unique approach underscores a sophisticated interdependence that is yet to be fully understood. Furthermore, NLRP3 appears to regulate cellular senescence so that its activation does not lead to pyroptosis. This suggests that senescent cells could be a persistent source of IL-1β production, thereby contributing to aging. The significance of NLRP3 in aging is highlighted by studies demonstrating that its knockout endows mice with a phenotype akin to HC, extending their HS and LS (Youm et al., 2013; Marín-Aguilar et al., 2020). Additionally, treatments targeting pathways upstream of NLRP3 have been shown to extend the HS of primates and reverse various aging symptoms in humans (Kumar et al., 2023; Singh et al., 2023). Therefore, NLRP3 is not merely a participant in the aging process but potentially acts as a master regulator. Modulating NLRP3 could significantly alter the health trajectories of individuals experiencing NLRP3-mediated accelerated aging. Since this process is largely driven by autologous components, the term ‘auto-aging’ is proposed. Further research is essential to understand the role of NLRP3 in accelerated aging entirely and to develop healthspan-extending therapies targeting this key regulator. A critical question remains: Should interventions aim to completely inhibit NLRP3 activation or selectively target specific activation pathways to maximize health benefits? While there appears to be redundancy among NLRs in defending against PAMPs, broad inhibition might increase susceptibility to specific infections by weakening primary defense mechanisms (Coll et al., 2016). Additionally, better biomarkers are needed to gauge the impact of such therapies on NLRP3 activity. Assessments often focus on free IL-1β levels in plasma, which are minimal due to neutralization by sIL-Rs. Alternatives could include measuring total IL-1β or utilizing surrogate markers like IL-6 downstream of IL-1β, which may provide a more accurate reflection of NLRP3-mediated inflammatory status.

Focusing on reversing age-related symptoms, as highlighted by the XPRIZE Healthspan competition, is an effective strategy to advance this emerging field. RCTs aimed at studying HS extension can be prohibitively expensive and lengthy and may also pose ethical challenges since they involve healthy participants. Conversely, RCTs that target the reversal of common aging symptoms can bypass many of these issues. This approach could catalyze the development of new treatments designed to mitigate the detrimental effects of aging, potentially leading to rapid advancements in the exciting and emerging field of health extension.

Lastly, will health extension be achieved through pharmaceutical treatments, or could it be managed with supplements? Pharmaceuticals are traditionally targeted at treating diseases and cannot be prescribed to healthy individuals. There are proposals to classify healthy people of a certain age as ‘*de facto*’ diseased to circumvent this issue. However, even if regulatory bodies accept this classification, the question remains: at what age should this apply? A conservative approach would likely set this age at 70 or older. For optimal effectiveness, healthspan-extending interventions might need to start earlier, which means that pharmaceutical treatments would be delayed until individuals are already experiencing a decline, potentially reducing their efficacy and benefits. Moreover, the costs of such treatments and the likelihood that insurers may not cover a universally applicable intervention are significant considerations.

Supplements, on the other hand, hold promise in addressing several of these challenges. They could be a viable solution, albeit with their own restrictions. Dietary supplements can be marketed to improve specific functions or to reduce disease risk factors, such as improving muscle strength or lowering cholesterol, which are considered health claims. However, making claims about reducing risks associated with aging will likely be challenging, as aging is not (yet) classified as a disease. Additionally, it is still being determined whether claims combining multiple benefits (e.g., improving muscle strength and cognition) can be substantiated and approved. For a supplement to carry a specific health claim, it must undergo development with the same rigor as a pharmaceutical, including obtaining pre-market approval. Moreover, if one or more bioactive ingredients have not been used previously in food, they must be developed and approved as novel foods to ensure they are non-toxic. However, these processes can be completed much faster and at significantly lower cost, resulting in more affordable product pricing. This affordability might help overcome the barrier of supplements being unlikely to be reimbursed.

Overall, the convergence of scientific breakthroughs and innovations is not just a trend, but a transformative force that is steadily bringing the prospect of exceptional health significantly closer. This exciting progress is not just a dream, but a reality that we are inching closer to. The ability to reverse aging symptoms in humans is seen as a crucial stepping stone toward the audacious goal of extending HS for everyone.
